# COVID-19 Vaccine Perception and Hesitancy Among Patients With Sickle Cell Disease in the Western Region of Saudi Arabia

**DOI:** 10.7759/cureus.21026

**Published:** 2022-01-08

**Authors:** Hamza Jan, Abdullah Waheeb, Hatem AlAhwal, Abdullah Almohammadi, Adel Al-Marzouki, Ahmed Barefah, Salem Bahashawan, Osman Radhwi

**Affiliations:** 1 Hematology, King Abdulaziz University Faculty of Medicine, Jeddah, SAU; 2 Hematology Research Unit, King Fahd Medical Research Center, Jeddah, SAU

**Keywords:** jeddah saudi arabia, covid-19 vaccination hesitancy, covid-19 vaccine, sickle cell disease: scd, covid-19

## Abstract

Background: In the era of the coronavirus disease-2019 (COVID-19) pandemic, the race toward shielding the public through vaccination is still going. Patients with sickle cell disease (SCD) require special consideration given their medical needs and the common side effects of immunization, affecting their decision. Therefore, we aimed to assess the perception and hesitancy toward COVID-19 vaccination in this population and explore the possible factors when it comes to vaccination decisions.

Methods: The present cross-sectional phone interview study was conducted between May 10 and 20, 2021. The questionnaire was administered by the medical staff. The participants were all patients with SCD presented to King Abdulaziz University Hospital in Jeddah, Saudi Arabia.

Results: Out of 346 patients, 147 patients agreed to participate. Only 52 (35.37%) patients received at least one dose of the nationally available vaccines, and there were no reported serious side effects. Among the unvaccinated participants, 45 patients (47.8%) were undecided. The most reported reasons for hesitancy were the fear of developing complications as their acquaintance had and the fear of developing brain blood clots post vaccination.

Conclusions: The number of vaccinated patients with SCD was unfortunately low in our study, secondary to hesitancy. This represents a significant barrier and needs to be tackled appropriately at any proper interaction with a patient with SCD. The absence of major side effects and vaso-occlusive crises is assuring.

## Introduction

The World Health Organization (WHO) has declared the coronavirus disease-2019 (COVID-19) a pandemic, which is caused by the novel “severe acute respiratory syndrome coronavirus 2” (SARS-CoV-2). This is the biggest epidemic since the previous century, and it is still spreading [1].

Until the 24th of May 2021, the number of confirmed cases worldwide has reached over 166 million cases, and confirmed deaths have exceeded 3.4 million [2]. In Saudi Arabia, the first reported case was on the 2nd of March, 2020 [3]. Moreover, more than 440,000 cases with approximately 7000 deaths have been reported till the 24th of May [4].

Sickle cell disease (SCD) is a genetic blood disorder caused by a mutation in the hemoglobin-beta gene on chromosome 11, which causes red blood cells to sickle, resulting in a sickle cell crisis and organ hypoxia [5]. From 2011 to 2015, the prevalence rate of SCD in Saudi Arabia was 48.34 per 1000 individuals [6]. 

Throughout the COVID-19 pandemic, patients with SCD were thought of as “high risk” due to impaired immunity, resulting from hyposplenism and systemic vasculopathy, which gives them the tendency for end-organ dysfunction and high risk for thrombosis [5,7]. In addition, previous studies have reported their vulnerability to acute viral illnesses [8] and subsequent exacerbation of vaso-occlusive crises and acute chest syndromes [9].

The secure-SCD registry was found to collect reported COVID-19 infections in SCD from eight different countries. For over a year since March 2020, it has registered 386 adults with proven COVID-19 infections where only 17% developed severe to critical COVID-19 infections and only 4.8% deaths related to COVID-19 [10].

There is no published experience regarding concerns and hesitancy for patients with SCD with the COVID-19 vaccines. Therefore, we aim to provide an insight for physicians on vaccinated patients with a better understanding when it comes to patient counseling. 

## Materials and methods

Study design

The study was designed as a descriptive cross-sectional survey from May 10 to 20, 2021. Data were collected via an interviewer-administered validated questionnaire (see supplementary appendix).

Inclusion and exclusion criteria

All patients with SCD above the age of 18 presented to King Abdulaziz University Hospital since 2015, numbering at 346, were enrolled. Medical students (H.J. and A.W.) contacted all 346 patients through phone interviews, given the paucity of physical clinical appointments during the pandemic.

Data synthesis

We asked about the side effects in vaccinated patients and the concerns and willingness regarding the COVID-19 vaccine in unvaccinated patients. Patients' demographic data were supplemented by the sickle cell registry data at King Abdulaziz University Hospital.

Data entry and analysis

For data entry, we used Microsoft Excel 2019, and for data analysis, Stata ver. 17.0 (StataCorp LLC, College Station, Texas, USA) was used. Summary statistics were described for all variables comparing the vaccinated group with the non-vaccinated group as well as comparing recipients of the Pfizer vaccine with recipients of the AstraZeneca vaccine. In addition, the reported outcomes between recipients of both vaccines were compared. Continuous variables were described as means with range. Categorical variables were described using a two-way table. The independent-sample t-test and Fisher’s exact test were used for both continuous and categorical variables, respectively. A p-value less than 0.05 was considered statistically significant.

Ethical approval and patient consent

The King Abdulaziz University Hospital research ethics committee approved this study (IRB 332-21), and it was carried out according to their guidelines and recommendations. The authors claimed verbal consent from each participant at the beginning of each phone interview.

## Results

Out of the 346 patients, 147 participated, 103 did not answer the call, 17 refused to participate, 38 had wrong phone numbers, and 41 were deceased. 

A summary of the participants is shown in Table 1. Out of the 147 participants, 20 (14%) were infected with COVID-19, and 8 out of the 20 (40%) were hospitalized due to COVID-19. The highest rates of infection were in September and October 2020. 

**Table 1:** Essential characteristics of the studied group COVID-19, coronavirus disease 2019.

		Vaccinated n = 53 (36.1%)	Not Vaccinated n = 94 (63.9%)	Total N = 147 (100%)	p-Value
Gender	Male	28 (52.8%)	51 (54.3%)	79 (53.7%)	1.0
	Female	25 (47.2%)	43 (45.7%)	68 (46.3%)	
Age mean (range)		32.2 (19-49)	30.4 (18-48)		0.15
Nationality	Saudi	36 (67.9%)	46 (48.9%)	82 (55.8%)	0.034
	Chadian	2 (3.8%)	10 (10.6%)	12 (8.2%)	
	Sudanese	3 (5.7%)	2 (2.1%)	5 (3.4%)	
	Yemeni	10 (18.9%)	34 (36.2%)	44 (29.9%)	
	Myanmarese	0 (0%)	1 (1.1%)	1 (0.7%)	
	Pakistani	2 (3.8%)	1 (1.1%)	3 (2.0%)	
Disease	Hb SS	33 (62.3%)	60 (63.8%)	93 (63.3%)	0.008
	Hb S Beta thalassemia	12 (22.6%)	32 (34.0%)	44 (29.9%)	
	Hb S trait	8 (15.1%)	2 (2.1%)	10 (6.8%)	
Documented COVID-19 infection	Yes	6 (11.3%)	14 (14.9%)	20 (13.6%)	0.623
	No	47 (88.7%)	80 (85.1%)	127 (86.4%)	
Hospitalization secondary to COVID-19 (n = 20)	Yes	2 (3.8%)	6 (6.4%)	8 (5.4%)	0.545
On hydroxyurea	Yes	25 (47.2%)	54 (57.4%)	79 (53.7%)	0.229
	No	28 (52.8%)	39 (41.5%)	67 (45.6%)	
	Unknown	0 (0%)	1 (1.1%)	1 (0.7%)	

**Figure 1:**
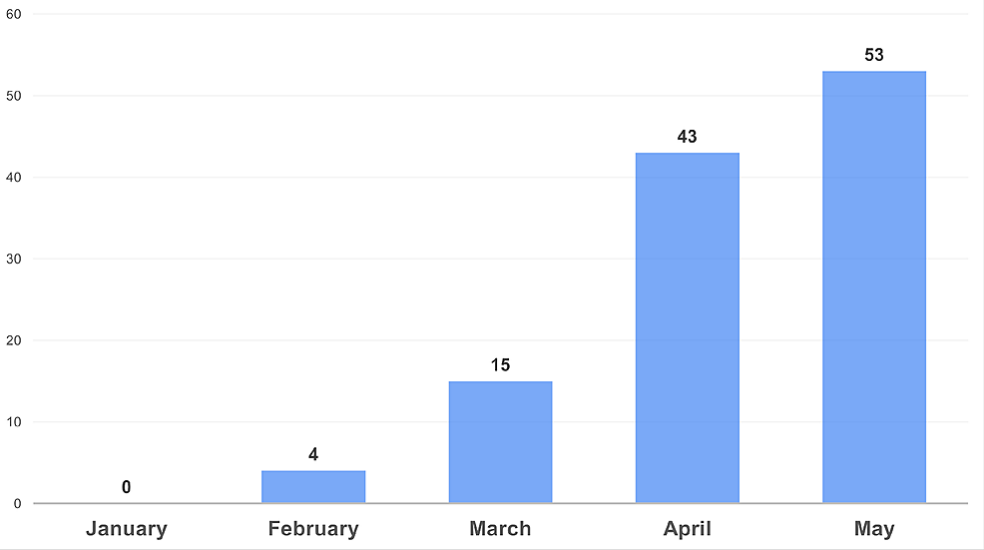
Cumulative number of vaccinated patients with sickle cell disease

Among the participants, 53 (36%) were vaccinated, with May 2021 witnessing the highest vaccination rate (Figure 1).

Most vaccinated participants received the BNT162b2-Pfizer vaccine (59.6%), while the other participants received the ChAdOx1 nCoV-19 Oxford-AstraZeneca vaccine (40.3%). Only 4 out of the 53 (7.5%) reported receiving the second dose. See Table 2 for further information regarding the vaccinated participants.

**Table 2:** Essential characteristics of the vaccinated group according to the vaccine type COVID-19, coronavirus disease 2019.

Total n = 52 Unknown = 1		Pfizer n = 31 (%)	AstraZeneca n = 21 (%)	p-Value
Gender	Male	14 (45.2%)	13 (61.9%)	0.27
	Female	17 (54.8%)	8 (38.1%)	
Age mean (range)		32.2 (21-49)	32.4 (19-47)	0.92
Nationality	Saudi	24 (77.4%)	12 (57.1%)	0.423
	Chadian	0 (0%)	1 (4.8%)	
	Sudanese	1 (3.2%)	2 (9.5%)	
	Yemeni	5 (16.1%)	5 (23.8%)	
	Pakistani	1 (3.2%)	1 (4.8%)	
Disease	Hb SS	20 (64.5%)	13 (61.9%)	0.463
	Hb S Beta thalassemia	5 (16.1%)	6 (28.6%)	
	Hb S trait	6 (19.4%)	2 (9.5%)	
Documented COVID-19 infection	Yes	3 (9.7%)	3 (14.3%)	0.675
	No	28 (90.3%)	18 (85.7%)	
Hospitalization secondary to COVID-19 (n = 6)	Yes	2 (6.5%)	0 (0%)	0.4
	No	1 (3.2%)	3 (14.3%)	
Injected with the second dose	Yes	3 (9.7%)	1 (4.8%)	0.486
	No	28 (90.3%)	19 (90.5%)	
On hydroxyurea	Yes	12 (38.7%)	13 (61.9%)	0.157
	No	19 (61.3%)	8 (38.1%)	

After the first dose, 42 (80.76%) of the vaccinated patients had mild side effects, with pain at the injection site being the most common (53.84%). Two vaccinated participants (3.84%), one from each vaccine group, had to present to acute hospital care from acquiring COVID-19 infection post vaccination, and only one participant (3.2%) developed a vaso-occlusive crisis. See Table 3 for a complete list of side effects reported by patients with SCD post vaccination.

**Table 3:** Reported outcomes post vaccination according to vaccine type

	Pfizer n = 31	AstraZeneca n = 21	Total N = 52	p-Value
Developed any complications	24 (77.4%)	18 (85.7%)	42 (80.76%)	0.721
Fever	5 (16.12%)	13 (61.9%)	18 (34.61%)	0.001
Pain at the site of injection	21 (67.74%)	7 (33.33)	28 (53.84%)	0.002
Headache	7 (22.58%)	9 (42.85%)	16 (30.76%)	0.21
Lethargy	4 (12.9%)	3 (14.28%)	7 (13.72%)	1.0
Diarrhea	0	1 (4.76%)	1 (1.92%)	0.429
Dizziness	2 (6.4%)	2 (9.52%)	4 (7.69%)	1.0
Chest pain	0	0	0	N/A
Shortness of breath	1 (3.22%)	1 (4.76%)	2 (3.84%)	1.0
Generalized pain	0	1 (4.76%)	1 (1.92%)	0.429
Chills	0	0	0	N/A
Nausea	0	0	0	N/A
Cough	0	1 (4.76%)	1 (1.92%)	0.429
Felt the need for analgesia or antipyretic medication	17 (54.8%)	16 (76.2%)	33 (63.34%)	0.149
Felt the vaso-occlusive crisis might occur	3 (9.7%)	6 (28.6%)	9 (17.3%)	0.133
Visited the emergency department post vaccination	1 (3.22%)	1 (4.76%)	2 (3.84%)	1.0

Most unvaccinated participants (n = 49, 52.1%) have registered but have not yet received the vaccine. However, some patients (n = 45, 47.8%) did not take the vaccine due to their fear of developing complications as their acquaintance had (42.22%) and the fear of developing brain blood clots (31.11%) post vaccination. See Table 4 for detailed reported concerns about the vaccine.

**Table 4:** Reported concerns by patients with sickle cell disease

Concern	Vaccinated (n = 10)	Not vaccinated (n = 35)	Total (n = 45)	p-Value
Brain blood clot	3 (30%)	11 (31.42%)	14 (31.11%)	0.38
Acquaintance with complications	3 (30%)	16 (45.71%)	19 (42.22%)	0.071
Fever	1 (10%)	3 (8.57%)	4 (8.88%)	1
Pain at site of injection	0	6 (17.14%)	6 (13.33%)	0.088
Allergy	0	1 (2.85%)	1 (2.22%)	1
Generalized pain	1 (10%)	0	1 (2.22%)	0.361
Breathing problems	2 (20%)	1 (2.85%)	3 (6.66)	0.295
Fear of drug interactions	0	1 (2.85%)	1 (2.22%)	1

After adjusting for age, gender, and disease type, no statistical differences were found when comparing the vaccinated and the unvaccinated participants (Table 1). The mean age was 32.2 and 30 (p = 0.15) for the vaccinated and the unvaccinated participants, respectively. The most common nationality was Saudi Arabian in both groups. The vast majority of vaccinated and unvaccinated individuals had HB SS type, 33 and 60, respectively. Only six vaccinated and 14 unvaccinated participants were previously infected with COVID-19, and only 8 (40%) out of the 20 infected patients required hospitalization with no intensive care unit admissions and were discharged home.

## Discussion

We aim to provide an insight for physicians while counseling patients with SCD regarding COVID-19 vaccination. In addition, the lack of data and information around the adverse effects of the COVID-19 vaccine meant that our study would answer questions that are vital to patients with SCD. No other studies were found to have tackled this issue, and here we are the first to report such side effects and explore patients’ hesitancy regarding the COVID-19 vaccine.

Saudi Arabia was one of the first countries to provide the vaccine to its residents voluntarily. No exclusions were made to their legal or health status. Vaccination first started in mid-December 2020 for senior and immunocompromised individuals and was extended to the whole population earlier in March 2021. Up to the last day in May, more than 14 million doses were given in all parts of Saudi Arabia. The Saudi government offered the vaccine to every resident in the country free of charge and an accessible registry online. Furthermore, the Ministry of Health launched more than 500 vaccination centers across the country for easier accessibility [11].

Our sample showed us that more patients had taken the Pfizer vaccine over the AstraZeneca vaccine. The fact that the Pfizer vaccine was approved by the Saudi Food and Drug Association (SFDA) before the AstraZeneca vaccine might have been a factor [12,13], albeit a minor one. The patients in our sample have consistently shown fear from the news surrounding the AstraZeneca vaccine and the possibility of having severe side effects such as blood clots [14]. This led patients to choose the vaccination centers equipped with the Pfizer vaccine despite the possible longer waiting time for appointments. 

Most of the concerned subjects (31.11%) feared “brain clots” after vaccination. This matter can improve by providing adequate knowledge and transparency regarding its occurrence and differentiating cerebral vein thrombosis from an ischemic stroke which is a known complication for patients with SCD [5]. In a large-scale local study, more than 1500 vaccinees were approached regarding side effects from the AstraZeneca vaccine. Fever was reported in 31.3%, pain at the site of injection in 30.5%, and musculoskeletal symptom in 27.5% [15].

The number of patients who acquired COVID-19 infection prior to their vaccination with mild outcomes contradicts the expected severe respiratory course the patients might experience. This has been reported in the published secure-SCD registry outcomes [10], which reported 17% requiring mechanical ventilation support in adults with SCD who acquired COVID-19 infection and also in the French experience where the rate was at 12.67% among adults aged more than 14 years [16].

Strengths and limitations 

Despite the advantages of this study in exploring and reporting the factors associated with the decision taken by the SCD patients, our study has limitations. First, we were unable to interview the participants in person due to COVID-19 restrictions; therefore, we conducted the interviews over the phone. Secondly, we did not explore the willingness of the participants to take the vaccine in the future, but instead, we asked if they had registered for a vaccine injection in the future. Finally, we only studied SCD patients who were managed at our hospital. This may lead to a potential selection bias in the results. 

## Conclusions

In conclusion, the majority of participants believed that vaccination’s adverse effects represent the most significant obstacle. However, we can overcome this by increasing public awareness and transparency regarding side effects and their prevalence. Moreover, most vaccinated participants did not develop significant side effects after vaccination, and the rate and type of their side effects were similar to vaccinated individuals with no SCD.

## Appendices

Interviewer-administered questionnaire:

Section 1:

*Did you **acquire** COVID-19 infection? YES/NO

If **hospitalized**: when and where?

*Did you **take **the COVID-19 vaccine? YES/NO (if no, go to section 2)

*What type of vaccine did you receive with your **first **dose? Pfizer/AstraZeneca

*Where and when was your **second **dose?

*What type of vaccine did you receive with your **second **dose? Pfizer/AstraZeneca

*Did you experience side effects after the **first **dose? Pain at site of injection/Fever/Headache/Dizziness/Shortness of breath/Limb swelling/Other:

*Did you experience side effects after the **second **dose? Pain at site of injection/Fever/Headache/Dizziness/Shortness of breath/Limb swelling/Other:

*Did you require pain medication or antipyretic, if febrile? NO/YES, mention

*Did you feel a vaso-occlusive crisis will occur after your vaccine dose? YES/NO

*Did you require a visit to the emergency department after the vaccine dose? YES/NO

Section 2

*Did you **book **for the vaccine dose? NO/YES

If yes, where and when?

*Do you have **concerns** about the vaccine? No/Fear of injection/Pain at site of injection/Fever/Complications occurred to acquaintances/ absence from work or school/Other

Section 3

*Currently, do you take **hydroxyurea**? YES/NO

*What is the best **contact method** for another encounter related to this research? Cellphone/home phone/WhatsApp/Email
